# Evidence for lateralized functional connectivity patterns at rest related to the tendency of externalizing or internalizing anger

**DOI:** 10.3758/s13415-022-01012-0

**Published:** 2022-05-24

**Authors:** Jennifer Consolini, Sara Sorella, Alessandro Grecucci

**Affiliations:** 1 Department of Psychology and Cognitive Science, University of Trento, Corso Bettini, 31, Rovereto, TN Italy; 2 Centre for Medical Sciences, CISMed, University of Trento, Trento, Italy

**Keywords:** Emotion, Anger, Functional connectivity, Prefrontal cortex, Prefrontal asymmetry

## Abstract

Previous electroencephalographic and brain stimulation studies have shown that anger responses may be differently lateralized in the prefrontal cortex, with outward-oriented responses (externalized anger) linked to left prefrontal activity, and inward-oriented responses (internalized anger) linked to right prefrontal activity. However, the specific neural structures involved in this asymmetry, and how they interact to produce individual differences, remain unexplored. Furthermore, it is unclear whether such asymmetry may be explained by general behavioral tendencies, known as Behavioral Activation and Behavioral Inhibition Systems (BIS/BAS). Therefore, we analyzed the tendency of externalizing and internalizing anger, respectively measured by the Anger-Out and Anger-In subscales of the State-Trait Anger Expression Inventory, with the patterns of functional connectivity at rest of 71 participants. A left, prefrontal, resting-state, functional connectivity pattern was found for externalizing anger (Anger-Out), including the left inferior frontal gyrus and the left frontal eye fields. By contrast, a right, prefrontal, resting-state, functional connectivity pattern was found for internalizing anger (Anger-In), including the rostral and lateral prefrontal cortex, the orbitofrontal cortex, the frontal pole, the superior, middle and inferior frontal gyri, and the anterior cingulate. Notably, these patterns were not associated with the BIS/BAS scores. In this study, for the first time, we provide evidence using fMRI functional connectivity for two specific lateralized circuits contributing to individual differences in externalizing and internalizing anger. These results confirm and extend the asymmetry hypothesis for anger and have notable implications in the treatment of anger-related problems.

## Introduction

Anger and its psychological mechanisms have been a topic of scientific interest for decades, although the specific neural bases that underlie the individual differences of this emotion remain a matter of debate (Alia-Klein et al., 2020; Gilam & Hendler, 2017; Sorella et al., 2021). Anger is an intense affective state, which may occur in situations encountered in the social context, starting from hostile cognitive interpretations attributed to external events (Bhave & Saini, 2009; De Panfilis et al., 2019; Gilam & Hendler, 2017; Grecucci et al., 2017; Potegal, 2010). Subsequently, it evolves into specific behavioral responses that vary according to individual differences and environmental factors (Bhave & Saini, 2009; Matsumoto et al., 2010; Wang et al., 2005). From a cognitive point of view, in humans, the motivation behind these reactions lies in the adaptive meaning that distinguishes anger: its function is to defend ourselves from the threats to our self-esteem, our sense of justice, our power and our ambitions (Aquino et al., 2006; Bies & Tripp, 2005). As a result of its self-preservation function, anger sometimes is expressed outwardly (externalized anger) with physical or verbal behaviors to face the anger-eliciting stimulus (Carver & Harmon-Jones, 2009; Kuppens et al., 2004; Smits & Kuppens, 2005). However, some individuals do not express anger outward but redirect it inward (internalized anger). In such cases, people do not face the provoking situation, but rather they internalize anger and often ruminate on the problem (Smits & Kuppens, 2005; Spielberger, 1999; Zinner et al., 2008).

To explain these two opposite styles of dealing with anger, some authors have hypothesized that the individual differences of externalizing or internalizing anger may be related to differences in approach and avoidance motivational systems (Carver & Harmon-Jones, 2009; Davidson, 1993a, 1993b, 1998, 2000; Smits & Kuppens, 2005; Wacker et al., 2003). In other words, some people are prone to express anger, because they are mainly driven by the approach system. Others are more inclined to internalize anger, because the avoidance system is predominant (Smits & Kuppens, 2005). This last option leads to the inhibition and the suppression of anger (Greenglass, 1996; Julkunen, 1996; Schwenkmezger & Hank, 1996; Smits & Kuppens, 2005; Smits et al., 2004).

Over the years, neuroscientists have tried to clarify the neural bases of externalizing or internalizing anger, providing some evidence of lateralized neural activity by using electroencephalography (EEG), transcranial direct current stimulation (tDCS), and transcranial magnetic stimulation (TMS) (Carver & Harmon-Jones, 2009; Harmon-Jones & Gable, 2018; Kelley et al., 2017).

In EEG studies, externalizing anger has been found to increase cortical activity in the left prefrontal cortex (PFC) (which is coherent with approach behaviors observed for positive emotions, see Carver & Harmon-Jones, 2009; Harmon-Jones & Gable, 2018; Kelley et al., 2017). On the other hand, internalizing anger (e.g., inhibiting and suppressing anger) was found to increase cortical activity in the right PFC (which is coherent with the avoidance tendencies associated with other unpleasant emotions) (Carver & Harmon-Jones, 2009; Harmon-Jones & Gable, 2018; Kelley et al., 2017). For example, Harmon-Jones & Sigelman (2001) found a correlation between aggression (reacting with increased anger) and the left prefrontal activity at rest after an anger-induction task (i.e., negative evaluation of a writing task). However, Zinner et al. (2008) showed that the degree of suppressing (internalizing) anger in an interracial context was associated with greater right electrical prefrontal activity (Harmon-Jones & Gable, 2018; Zinner et al., 2008).

Another line of evidence of this asymmetry was provided by experiments that modulated the activity of the PFC with tDCS during anger-eliciting tasks. Hortensius et al. (2012) found that anodal tDCS stimulation over the left dorsolateral prefrontal cortex (dlPFC) increased perceived anger and aggressive behaviors during the Taylor Aggressive Paradigm (TAP). Inversely, as pointed out in an interesting review on the prefrontal asymmetry (Kelley et al., 2017), Dambacher et al. (2015) demonstrated that the anodal stimulation of the right dlPFC during the TAP decreased aggressive behaviors. Moreover, the tDCS induced-increased activity of the right dlPFC was associated with increased rumination on the angering event, compared with both left dlPFC and sham stimulation (Kelley et al., 2013). This study provided evidence for the association between internalizing anger, rumination, and right prefrontal activity (Kelley et al., 2013).

Similar results were obtained by using TMS. The interference of the right dlPFC activity induced by TMS showed a lower tolerance of provocative stimuli and an increased tendency to respond to those stimuli through approach-oriented responses, as a result of the left hemisphere predominance over the right one (d’Alfonso et al., 2000). The opposite result was obtained when stimulating the left dlPFC (d'Alfonso et al., 2000). Overall, these results converge on the idea of a prefrontal asymmetry for externalizing and internalizing anger.

However, one major limitation of the previous studies is that they rely on techniques that do not allow for an understanding of the exact neural structures involved in externalizing or internalizing anger. Indeed, it is still not clear whether the brain regions stimulated in tDCS-TMS studies are part of larger circuits and, if this is the case, how these regions interact to produce opposite anger tendencies.

Functional magnetic resonance imaging (fMRI) allows examination of functional connectivity among regions. Although fMRI studies have not probed prefrontal asymmetry specifically, there have been studies using both task- and resting-state fMRI to examine neural correlates more broadly underlying of anger, experiences of anger (Sorella et al., 2021), its control (Alia-Klein et al., 2020; Sorella et al., 2022), or individual predisposition to experience anger (Fulwiler et al., 2012; Romero-Martìnez et al., 2019). Interestingly, a review of fMRI studies showed that the experience of anger relies on bilateral activations of the ventrolateral prefrontal cortex and the anterior insula (Sorella et al., 2021); in addition, when considering network-based rs-fMRI, the DMN was associated not only with anger control (Sorella et al., 2022) but also with anger mentalization (Alia-Klein et al., 2020). In particular, medial prefrontal brain regions seem to be involved in anger control abilities (Alia-Klein et al. 2020). Also, rs-fMRI connectivity based on regions-of-interest (ROIs) showed that the connections between frontal brain regions and subcortical areas are inversely associated with trait anger and positively associated with anger control (Fulwiler et al., 2012; Romero-Martìnez et al., 2019). However, these studies did not explicitly investigate prefrontal asymmetry patterns associated with anger; furthermore, the majority of them were conducted relying on different paradigms, such as interpersonal games or imaginative techniques, which prevented consistent findings on the neural correlates of anger (see this meta-analysis of Sorella et al., 2021, for a summary on fMRI studies on anger).

Another limitation of previous studies is that they did not take into account individual differences in expressing anger, because they were mainly based on average task performance. Moreover, the low level of replicability of behavioral and neuroimaging task-based acquisition protocols has been recently underlined (Elliott et al., 2020). Even if reliability of univariate measures has been addressed in both task-based and connectivity fMRI studies (Noble et al., 2021; Taxali et al., 2021), when considering between-subjects research on individual-differences in human traits, rs-FC may be preferable. Indeed, common task-based fMRI measures are robust when considering differences between conditions (e.g., target vs. neutral stimuli), but they are less suitable when considering differences between people (Elliott et al., 2020). Indeed, task-based affective procedures involve a relatively limited duration of emotional states and are useful for assessing state-like characteristics, whereas emotional traits questionnaires represent more stable measures that can be associated with rs-FC (Fulwiler et al., 2012; Angelides et al., 2017). Therefore, we decided to take into account resting-state functional connectivity (rs-FC) to see whether it may be associated with stable personality traits related to anger expression. Importantly, such approach may not only overcome the limited duration of task-based fMRI studies, which is more useful for assessing state-like rather than trait-like characteristics, but it also may shed light on the specific brain regions involved in the individual differences in externalizing and internalizing anger. Indeed, as opposed to EEG and stimulation techniques, rs-FC allows obtaining information about the areas involved and how they interact, by determining the nature of changes in the metabolic activity occurring on the same temporal level between different voxels (Friston, 2011). Self-report data can then be used to discover the specific rs-FC patterns associated with the individual differences in different anger tendencies. As far as we know, this is the first attempt to apply rs-FC analyses to study the individual differences in externalizing and internalizing anger.

In light of this, the main purpose of the current research is to conduct an rs-FC analysis to test the hypothesis that the individual differences in externalizing anger are associated with specific left lateralized frontal FC patterns, whereas internalizing anger should be associated with more right frontal FC patterns. To study the tendency to express or internalize anger, we employed the Anger-Out and Anger-In subscales of the State-Trait Anger Expression Inventory (STAXI), a largely validated instrument proposed by Spielberger (1999). In line with previous results based on different methods, we predict that externalizing anger, as part of the approach motives, will be associated with left rs-FC patterns (Hewig et al., 2004). More specifically, we expect to find a clear involvement of the left dlPFC, a region known to be associated with angry responses to provoking situations (d’Alfonso et al., 2000; Hortensius et al., 2012). By contrast, we predict to find evidence of a right rs-FC pattern for internalizing anger in line with previous findings (Zinner et al., 2008; but see Hewig et al., 2004; Wacker et al., 2003 for discrepant results). In particular, we expect the involvement of the right dlPFC (Dambacher et al., 2015; Kelley et al., 2013) but also for the right ventrolateral prefrontal cortex (vlPFC), previously associated with the inhibition of angry reactions in a violent video game exposure (Riva et al., 2015). Notably, the vlPFC also has been associated with rumination, a usual consequence of internalized anger (Denson et al., 2009; Fabiansson et al., 2012; Sorella et al., 2021).

Moreover, to assess the possibility that results may be at least partially explained by the Behavioral Activation/Inhibition systems (Sutton & Davidson, 1997; Watson et al., 2016), we tested the hypothesis that the rs-FC patterns could be associated with BIS/BAS scales (Carver & White, 1994). The BIS/BAS questionnaire measures avoidance behaviors (Behavioral Inhibition System, BIS scale) and approaching behaviors (Behavioral Activation System, BAS scale) (Carver & White, 1994; Leone et al., 2002). Authors suggested that individual differences in these two dimensions are associated with people reactions to provocative stimuli, facing them or withdrawing from them (Gray, 1990; Heponiemi et al., 2003; Smits & Kuppens, 2005). In partial support of this relation between BIS/BAS and externalizing or internalizing anger, previous studies demonstrated that BAS scores positively correlate with increased left frontal cortex activity (Amodio et al., 2008; Coan & Allen, 2003; Gable & Poole, 2014; Harmon-Jones & Allen, 1997; Sutton & Davidson, 1997; Watson et al., 2016). In contrast, greater right activity was associated with BIS scores (Sutton & Davidson, 1997; Watson et al., 2016). Building on the above consideration, the second purpose of this paper was to understand whether the frontal asymmetry for anger expression styles also might be related with the Behavioral Activation and Inhibition Systems. One hypothesis is that the circuit involved in externalizing and internalizing anger overlap with the Behavioral Activation and Inhibition Systems. If this is the case, the same left lateralized circuit may be associated with both Anger-Out and BAS scores, and the same right lateralized circuit may be associated with both Anger-In and BIS scores. However, an alternative hypothesis is that the two constructs are independent, and the observed asymmetry in rs-FC patterns is specific for anger.

## Materials and methods

### Participants

For our study, we capitalized on the data derived from the MPI-Leipzig Mind Brain-Body dataset (OpenNeuro Dataset, http://openneuro.org, RRID:SCR_005031, accession number ds000221), which was made available by an extended cross-sectional project conducted at the Max Planck Institute (MPI) of Human Cognitive and Brain Sciences in Leipzig, Germany and approved by the ethics committee of the University of Leipzig (097/15-ff) (Babayan et al., 2018; Mendes et al., 2019). This open-access database comprises behavioral and MRI data from a sample of 318 participants. Participants were recruited through online and poster advertisement and had to provide informed consent to participate in the project. Furthermore, they were informed of a monetary refund obtainable at the end of the research. Before the effective participation in the research project, subjects were screened for past and present psychiatric and neurological disorder and had to be considered medically eligible to undergo magnetic resonance sessions. Also, they had to fulfill the MRI safety requirements of the MPI-CBS (Mendes et al., 2019). For the current research, 71 healthy subjects (42 males) with mean age of 26.02 (±3.53) were selected on the basis of the following criteria: availability of both structural and resting-state magnetic resonance imaging (rs-fMRI) images, age between ages 20 and 40 years (since in the dataset age was reported in a 5-year interval, we used the midpoint for calculations), and availability of the STAXI and BIS/BAS questionnaires’ scores (see next section for more details).

### Behavioral data

To study the individual differences behind externalizing or internalizing anger, the scores from the Anger-Out and Anger-In scales of the STAXI (Spielberger, 1999) were taken into account. Anger-Out measures the inclination to direct anger outwards (e.g., externalizing anger) through actions or language that can be negative and poorly controlled. On the other hand, Anger-In measures the tendency to internalize anger and to suppress or inhibit it (Spielberger, 1999). We considered these scales from the 44-item State-Trait Anger Expression Inventory (Spielberger, 1988), both the Anger-In and Anger-Out scales, ranging from a minimum score of 8 to a maximum score of 32, consist of 8 items rated on a 4-point Likert scale (from 1, strongly disagree, to 4, strongly agree) (Fuqua et al., 1991). In this sample, the mean score for Anger-In was of 15.87 (±4.32) and for Anger-Out was of 12.03 (±3.1).

To measure differences in motivational systems, the BIS/BAS questionnaire was taken into account. This questionnaire has been developed to measure the individual differences in the sensitivity of two neurological systems in response to relevant environmental cues; the first (BIS) regulates aversive motivation, whereas the second (BAS) regulates appetitive motivation (Carver & White, 1994). In particular, the BIS scale (7 items) investigated the motivational incentives behind behavioral avoidance and inhibition, such as concern about the possibility that something risky may happen. Conversely, the BAS scale measures the reward sensitivity and the desire to achieve goals, from which we activate approach behaviors. The BAS score was the sum of three subscales: BAS Drive (4 items); BAS Fun Seeking (4 items); and BAS Reward Responsiveness (5 items). Each item of the questionnaire is evaluated on a 4-point Likert Scale (from 1 – strongly disagree to 4 – strongly agree). BIS ranges from 7 to 28 while BAS (sum of Drive, Fun Seeking, and Reward) ranges from 13 to 52. In this sample, the mean score of BIS was 20.62 (±2.95), and the mean score of BAS was 37.69 (±3.73).

To check whether Anger-Out and Anger-In were respectively associated with BAS and BIS scores, we performed correlations. While no significant relationship emerged between Anger-In and BIS (r = 0.12, *p* = 0.319), a significant relationship emerged between Anger-Out and BAS (r = 0.25, *p* = 0.035).

### MRI data acquisition

A high-resolution structural scan and four rs-fMRI scans were collected on a 3T Siemens Magnetom Verio scanner (Magnetom Verio, Siemens Healthcare, Erlangern, Germany) for each participant (Mendes et al., 2019). Two gradient echo field maps, two pairs of spin echo images with reversed phase encoding direction, and a low-resolution structural image using a FLAIR sequence for clinical screening were additionally captured (Mendes et al., 2019). The structural – T1-weighted – acquisitions were obtained using the MP2 RAGE sequence (Marques et al., 2010), with the following parameters: TR = 5000 ms; TE = 2.92 ms; TI1 = 700 ms; TI2 = 2,500 ms; flip angle_1_ = 4°; flip angle_2_ = 5°; voxel size = 1.0-mm isotropic; duration = 8.22 min (Mendes et al., 2019). The rs-fMRI images were recorded utilizing GE-EPI sequence using these criteria: voxel size = 2.3-mm isotropic; FOV = 202 x 202 mm^2^; imaging matrix = 88 x 88.64 slices with 2.3-mm thickness; TR = 1,400 ms; TE = 39.4 ms; flip angle = 69°; echo spacing = 0.67 ms; bandwidth = 1,776 Hz/Px; partial Fourier 7/8; no prescan normalization; multiband acceleration factor = 4.657 volumes; duration = 15.30 min (Mendes et al., 2019).

### Pre-processing

All images were pre-processed with CONN toolbox (Connectivity Toolbox, https://www.nitrc.org/projects/conn, RRID:SCR_009550) running through MatLab R2019a (MATLAB, http://www.mathworks.com/products/matlab/, RRID:SCR_001622, MathWorks, Inc., Natick, MA) and SPM12 (SPM, https://www.fil.ion.ucl.ac.uk/spm/software/spm12/, RRID:SCR_007037) software. The default pipeline was executed with the following steps: functional realignment and unwarping, translation and centering, functional outlier detection (we applied the conservative settings, which use 0.5-mm and 3 SD thresholds, rather than the “liberal” setting, which use 2-mm and 9 SD thresholds), functional direct segmentation and normalization (2-mm resolution), structural translation and centering, structural segmentation and normalization (2-mm resolution), functional and structural smoothing (spatial convolution with Gaussian kernel 8 mm, which is set by CONN as default value). Then, factors that are potential confounds are estimated and removed for each voxel and for each subject relying on Ordinary Least Squares (OLS) regression. An anatomical component-based noise correction procedure (aCompCor) was run during the denoising step; in this way, the artefactual sources generated from head movements or physiological effects (e.g., respiration rate) were removed. In particular, noise components from cerebral white matter and cerebrospinal areas (5 regressors each), estimated subject-motion parameters (12 regressors defined from the estimated subject-motion parameters to minimize motion-related BOLD variability: 3 translation and 3 rotation parameters plus their associated first-order derivatives) and identified outlier scans or scrubbing (removal of volumes) were considered and removed. Finally, the quality of the data was checked through Quality Assurance plots showing the homogeneity of the global activation.

### Functional connectivity analyses

**Fig. 1:**
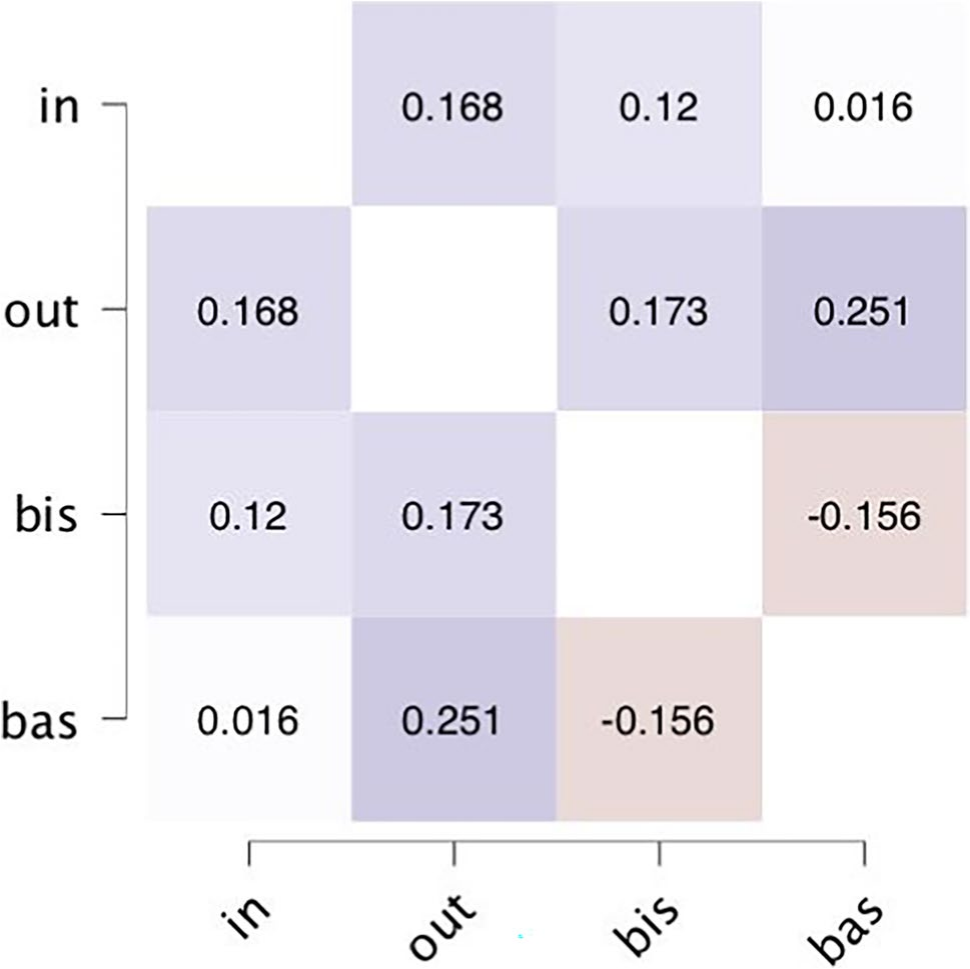
Correlation Matrix of Anger-In, Anger-Out, BIS, and BAS Scores

To examine the rs-FC across multiple brain regions in relation to the behavioral data, a Multivariate ROI-to-ROI Connectivity (mRRC) matrices analysis was conducted using CONN toolbox. This analysis was adopted to study multivariate models predicting each voxel BOLD signal from all the ROIs simultaneously. Building on the previous literature showing an involvement of different ventral, dorsal, and medial prefrontal regions in anger processes (Beyer et al., 2015; Denson et al., 2009; Alia-Klein et al., 2020; Sorella et al., 2021, 2022), we included all the bilateral frontal regions among the set of predefined ROIs considered by CONN to test the hypothesis of a frontal asymmetry: inferior frontal gyrus (IFG); lateral PFC; frontal eye field (FEF); rostral PFC, anterior cingulate (AC); medial PFC; IFG pars opercularis and pars triangularis; middle frontal gyrus (MFG); superior frontal gyrus (SFG); frontal pole (FP); orbitofrontal cortex (OFC); frontal operculum (FO); and anterior cingulate cortex (ACC). CONN’s default atlas—FSL Harvard-Oxford Atlas—generated the set of ROIs (Whitfield-Gabrieli & Nieto-Castanon, 2012). Mean time-series of each ROI were extracted from the pre-processed data. Subsequently, a multivariate ROI-to-ROI analysis was performed in CONN, where semipartial correlations were computed between the time courses of the selected ROIs of both hemispheres, to identify the connections between each pair of ROIs after discounting effects that may be mediated or accounted for by other ROIs. Correlation coefficients underwent Fisher’s r-to-z transformation to ensure the normality of the distribution. Therefore, during these steps of the first level analysis, the degrees of association (rs-FC values) between each pair of regions per subject were extracted. Then, to obtain an account of the connectivity association with the STAXI behavioral measures, the relationship between the scales’ scores and the rs-FC values was evaluated in the second-level analysis. To this aim, a multiple regression was performed as second level analysis to examine the relation between the rs-FC values previously obtained and the Anger-Out, Anger-In, and BIS/BAS scales’ scores. Since two scales were not normally distributed (i.e., Anger-In and Anger-Out), before the second-level analysis we transformed the values in z-scores. In addition, given a significant correlation between Anger-Out and BAS scores (Fig. 1), we decided to perform two different multiple regression; given this double analysis, we considered results as significant with a *p*-value < 0.025 (Bonferroni correction for the two regressions). Then, also gender and age variables were further considered, in order to control for their influence in the analyses. Results were considered significant at a connection-level threshold of *p* < 0.025 false discovery rate (FDR) corrected. CONN software was also used to visualize ROI-to-ROI connectivity, while JASP was used for the correlation matrix (https://jasp-stats.org).

## Results

### Brain activity

**Fig. 2:**
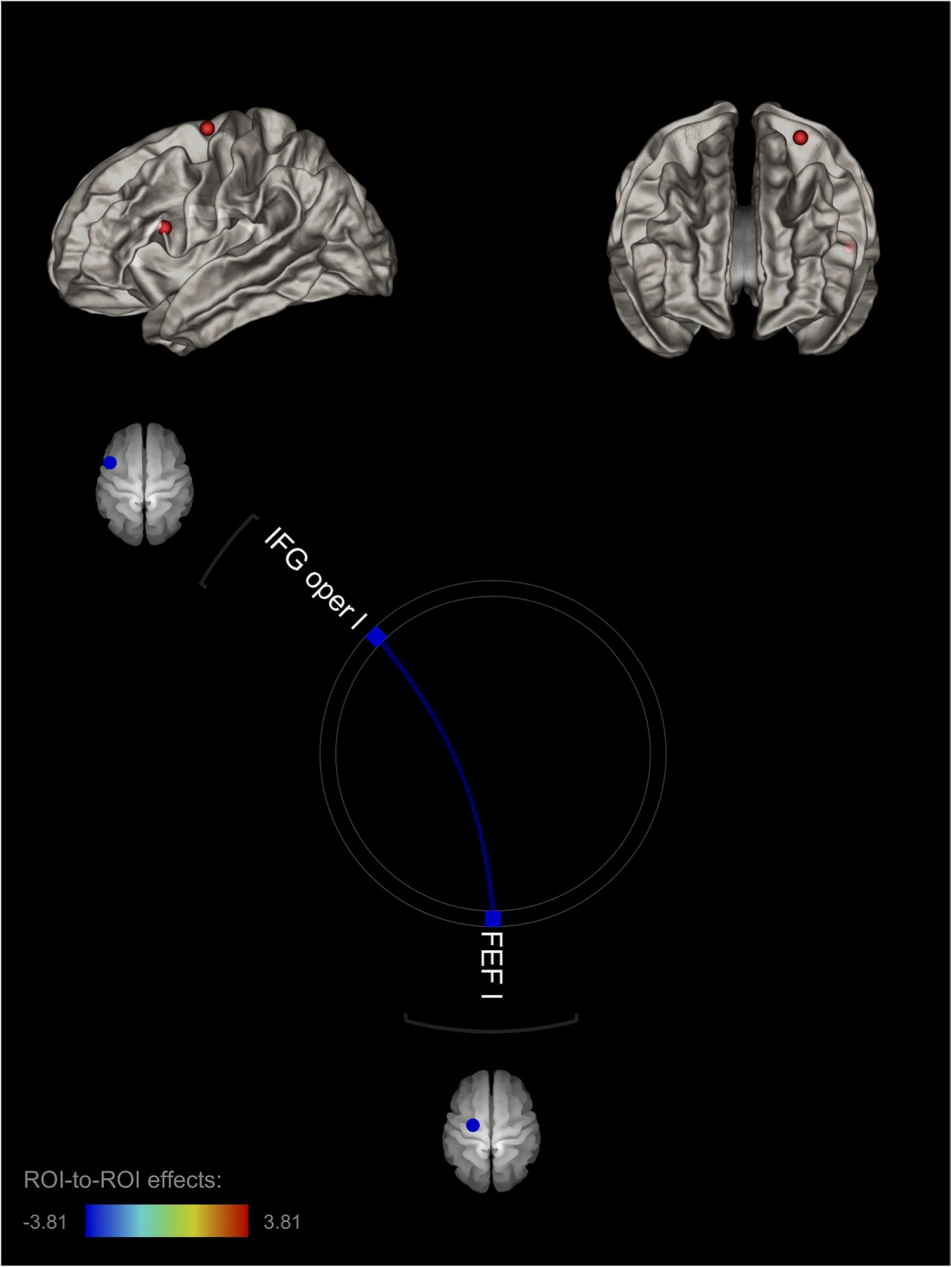
Left-lateralized, connectivity pattern associated with Anger-Out. Note. The left-lateralized neural pattern of rs-FC associated with the externalization of anger (Anger-Out) included the left IFG (IFG oper l) and the left FEF (FEF l)

As predicted, a left frontal rs-FC pattern was significantly associated with Anger-Out, whereas a right frontal rs-FC pattern was significantly associated with Anger-In. Specifically, Anger-Out was significantly associated with a negative rs-FC pattern, including the left IFG pars opercularis and the left FEF (T(68) = −3.81; *p*-FDR = 0.008) (part of the vlPFC and dlPFC respectively; Fig.2; Table 1). In particular, there was a negative relationship with anger externalization, such that as the Anger-Out scores increased, the negative connectivity between these regions decreased.

**Table 1:** Brain connectivity associated with Anger-Out and Anger-In scores

Connectivity	No effect of anger		With additional effect of anger		
	T(70)	*p*-FDR*	Scale	T(68)	*p*-FDR*
IFG oper (l) – FEF (l)	4.38	<0.001	ANGER-OUT	-3.81	0.008
RPFC (r) –FP (r)	20.57	<0.001	ANGER-IN	-4.29	0.008
RPFC (r) – OFC (r)	19.30	<0.001	ANGER-IN	-4.26	0.008
RPFC (r) – IFG tri (r)	-4.78	<0.001	ANGER-IN	-3.52	0.005
RPFC (r) –SFG (r)	12.02	<0.001	ANGER-IN	-3.50	0.005
RPFC (r) – MFG (r)	11.45	<0.001	ANGER-IN	-3.31	0.006
RPFC (r) – IFG (r)	-4.55	<0.001	ANGER-IN	-3.30	0.006
RPFC (r) – LPFC (r)	12.36	<0.001	ANGER-IN	-3.21	0.007
RPFC (r) – IFG oper (r)	11.84	<0.001	ANGER-IN	-2.93	0.015
LPFC (r) - ACC	3.00	0.006	ANGER-IN	-3.91	0.005
LPFC (r) - RPFC (r)	12.36	<0.001	ANGER-IN	-3.21	0.017
AC - LPFC (r)	0.16	0.877	ANGER-IN	-3.62	0.010
AC - FP (r)	10.21	<0.001	ANGER-IN	-3.53	0.010
AC – MFG (r)	1.27	0.246	ANGER-IN	-3.16	0.020

For each ROI-to-ROI connection, results on the connectivity without and with effects of anger are reported. In the first case, positive and negative values of T(70) represent positive and negative connectivity between each couple of regions. In the second case, negative values of T(68) represent the negative relationship between the anger scale (Anger-Out or Anger-In) and the specific ROI-to-ROI connectivity.

**Fig. 3:**
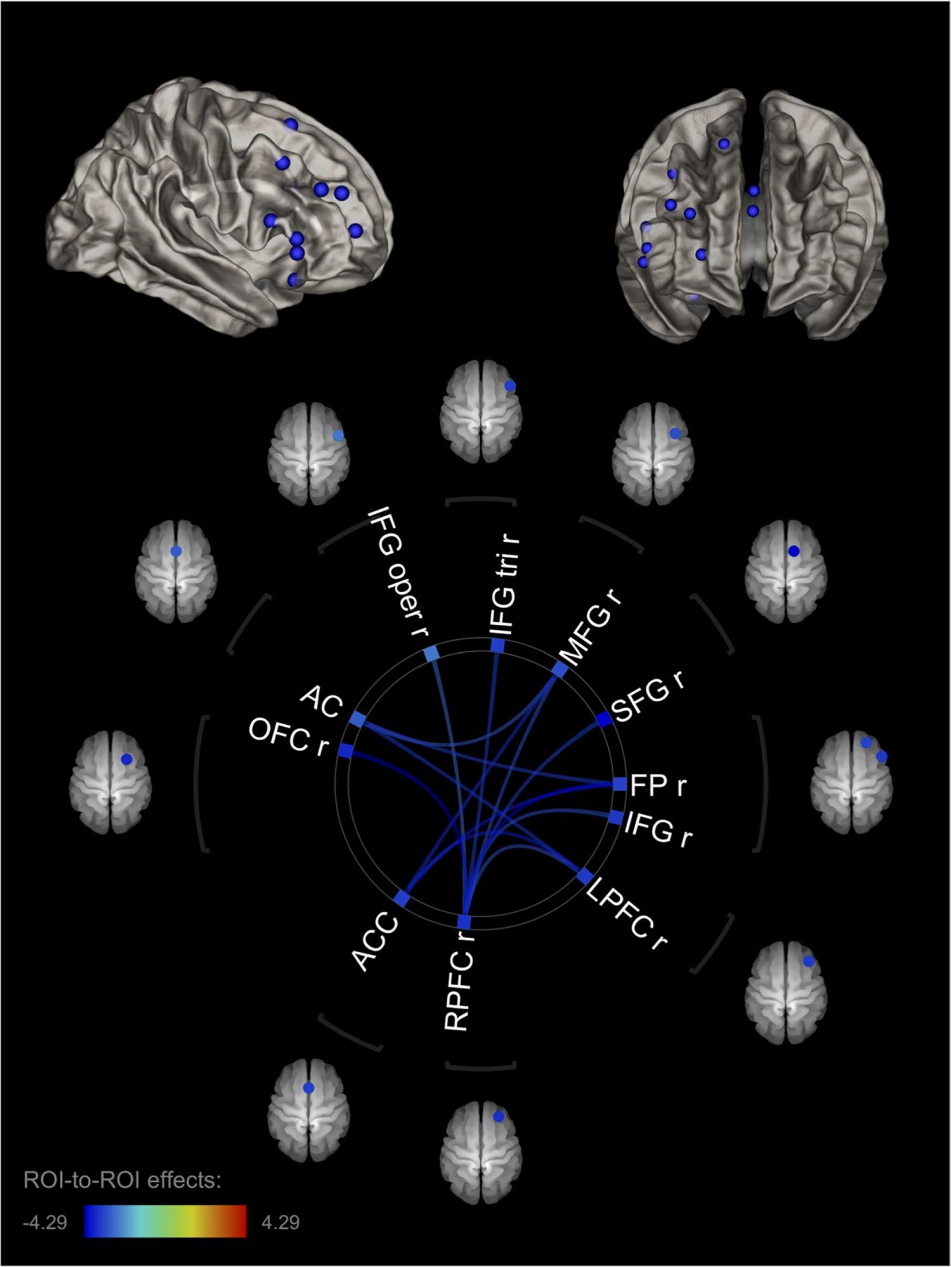
Right-lateralized connectivity pattern associated with Anger-In. Note. The right lateralized connectivity pattern associated with the inwardly expression of anger (Anger-In) included the right rostral (RPFC r) PFC, the right lateral PFC (LPFC r), the right OFC (OFC r), the right FP (FP r), the right superior (SFG r), middle (MFG r), and inferior frontal gyri (IFG r; IFG oper r; IFG tri r) and the AC

Moreover, several rs-FC patterns were significantly associated with Anger-In, including right lateralized regions of the dlPFC and vlPFC (Fig. 3; Table 1). Specifically, Anger-In was significantly associated with negative functional connectivity between the right rostral PFC and the right FP (T(68) = −4.29; *p*-FDR = 0.008), the right OFC (T(68) = −4.26; *p*-FDR = 0.008), the right IFG pars triangularis (T(68) = −3.52; *p*-FDR = 0.005), the right SFG (T(68) = −3.50; *p*-FDR = 0.005), the right MFG (T(68) = −3.31; *p*-FDR = 0.006), the right IFG (T(68) = −3.30; *p*-FDR = 0.006), the right lateral PFC (T(68) = −3.21; *p*-FDR = 0.007), and the right IFG pars opercularis (T(68) = −2.93; *p*-FDR = 0.015). Anger-In also was significantly associated with negative functional connectivity between the right lateral PFC and the ACC (T(68) = −3.91; *p*-FDR = 0.005), the AC (T(68) = −3.62; *p*-FDR = 0.007), and the right rostral PFC (T(68) = −3.21; *p*-FDR = 0.017); scores of Anger-In also were associated with negative functional connectivity between the ACC and the right FP (T(68) = −3.92; *p*-FDR = 0.003), the right lateral PFC (T(68) = −3.91; *p*-FDR = 0.003) and the right MFG (T(68) = −3.75; *p*-FDR = 0.003); finally, Anger-In was associated with negative functional connectivity between the AC and the right lateral PFC (T(68) = −3.62; *p*-FDR = 0.010), the right FP (T(68) = −3.53; *p*-FDR = 0.010), and the right MFG (T(68) = −3.16; *p*-FDR = 0.020). In particular, all of these rs-FC patterns were characterized by negative relationships with anger internalization, such that as the Anger-In scores increased, the negative connectivity between these regions decreased.

When considering gender and age in the regression, all relationships were still significant except for the connections associated with Anger-In between the AC and the right MFG (T(66) = −2.97; *p*-FDR = 0.035). See Fig. 3 and Table 1 for more details. No significant rs-FC pattern for the BIS/BAS scales was visible (*p*-FDR < 0.025).

## Discussion

The purpose of this study was to provide evidence for specific lateralized connectivity patterns associated with individual differences in externalizing and internalizing anger. Previous studies suggested that left frontal electrical activity is associated with externalizing anger, and right prefrontal cortical activity with internalizing anger. However, due to methodological limitations, previous studies could not provide a clear connectivity map of the exact brain structures associated with these opposite anger tendencies. By using rs-FC analyses, we identified for the first time the specific brain regions connected in two lateralized circuits and associated with the tendencies to externalize and internalize anger. To study these individual differences, we relied on the Anger-Out and Anger-In STAXI scales. Higher scores for the Anger-Out scale indicate a tendency to display angry reactions toward anger provoking situations, or in other words, to externalize anger. By contrast, higher scores for the Anger-In scale indicate a tendency to redirect anger inward, or to suppress it, or in other words to internalize it. We found a left prefrontal negative connectivity pattern between the left IFG pars opercularis (part of the vlPFC) and the left FEF (part of dlPFC) associated with Anger-Out. In particular, as Anger-Out scores increased, this negative connection decreased. In contrast, a larger pattern of right prefrontal negative connectivity including the right rostral and lateral PFC, as well as the right OFC, the right inferior, middle and superior FG (collectively part of the dlPFC), and the AC, was associated with Anger-In. In particular, as Anger-In scores increased, these negative connections decreased. Notably, no prefrontal connectivity pattern was associated with BIS/BAS scale, thus making our hypothesis about the prefrontal asymmetry implication in anger externalization and internalization, and not in approach or avoidance, supported by these analyses. In what follows, we discuss in detail our main findings.

The negative functional connectivity between the left IFG (pars opercularis) and the left FEF was associated with the Anger-Out scale. The left IFG is strongly related with anger experience in normal (Sorella et al., 2021) and in individuals with high levels of trait anger (Tonnaer et al., 2017). The IFG, being a part of the ventral premotor cortex, also may play a role in complex and goal directed actions’ planning (Binkofski & Buccino, 2006), which is a specific feature of externalizing anger to reach the obstructed relevant goal (Dollard et al., 1939; van Doorn et al., 2014). The FEF plays a key role in both visual attention and perceptual oculomotor coordination (Brooks & List, 2006). Therefore, it is evident that the connectivity pattern between these two regions may be associated with the experience and the following attentional/motoric control involved in externalizing anger. Indeed, from an evolutionary point of view the approach-motivated individual is inclined to redirect attention toward goal relevant stimuli and initiate actions to obtain such goals (Matsumoto et al., 2010). Anger may increase both directed attention toward relevant objects and action planning to reach the goal. If this is true, our results are in line with the role of IFG and FEF in contributing to the so-called hostile attentional bias (Sorella et al., 2021; Walters et al., 2016) linked to the experience of anger. In particular, our results show that as anger externalization decreases, the connectivity between these areas increases. This could mean that attentional and goal directed processes associated with these regions could be characterized by a reciprocal interference that increases as the externalization of anger increases; for example, it has been found that FEF and inferior frontal regions are both involved in top-down attention, but the second is more context dependent (Bedini & Baldauf, 2021). A hypothesis could be that the processes associated with FEF and IFG could compete when associated with anger externalization. In addition, these areas associated with anger externalization could play an inhibitory role, meaning that higher values of this tendency would be associated with a lower inhibition of the expression of anger. Indeed, not only the right IFG but also the left IFG has been associated with the inhibition of response (Swick et al., 2008). Following these observations, a possible explanation of our results might imply the inhibitory role of the left IFG on the left FEF, reduced as anger externalization scores increase. On the other hand, the possible inhibitory role of the FEF should be considered too, as previous results indicate a role in the inhibition of perceptual information (Smith et al., 2005). However, literature evidence is still lacking on this topic, and further studies are needed to better clarify these points in particular when considering anger and affective reactions. Indeed, previous methods, such as EEG and stimulatory techniques rely on different brain measures. Nevertheless, our results confirm that Anger-Out modulates the strength of left prefrontal brain connectivity.

Regarding the brain connectivity associated with the Anger-In scale, different patterns of right prefrontal negative connectivity emerged, involving the rostral and lateral PFC, the OFC, the FP, the SFG, the MFG, the IFG, and the AC. Of these regions, the right ventrolateral parts of the PFC (including the IFG) are involved not only in anger conceptualization (Sorella et al., 2021) but also with ruminative processes (Fabiansson et al., 2012; Gilam et al., 2017), which is one of the side effects of internalizing anger. More specifically, Sorella and collaborators (2021) proposed that the activity of the right IFG can be linked to a conceptual elaboration of anger that can elicit a modulation of cognition (e.g., reappraisal or rumination) or behavior (e.g., inhibition), both of them characteristic of internalizing anger. Also, the IFG, is usually associated with further emotional processing to regulate emotions or reframe the emotional stimulus, such as emotional labeling (Lieberman et al., 2007; Lieberman, 2011), interpersonal reappraisal (Grecucci et al., 2013a, b, c) and top-down regulation of the amygdala (Morawetz et al., 2016). When internalizing anger, these processes may be engaged to help the individual to reduce or inhibit anger. Moreover, the IFG is linked to response inhibition (Aron et al., 2014) and may play an important role to block the behavioral expression of anger, or in other words, the externalization of anger. The dlPFC (including the SFG and the MFG) may be involved not only in reappraising the angry stimulus and eventually in implementing emotion regulation strategies (Buhle et al., 2014; Grecucci et al., 2013b, c; Messina et al., 2021; Ochsner & Gross, 2005) but also in directly suppressing anger expression, as shown by tDCS studies in which the stimulation of the right dlPFC mitigates aggressiveness and negative emotional responses (Dambacher et al., 2015). The OFC is similarly involved in emotion regulation, but it seems to have a key role in anger control for its mediatory role in guiding insula’s activity when anger is experienced (Seok & Cheong, 2019; Sorella et al., 2021). In addition, alterations of the OFC-amygdala connectivity in clinical populations are associated to aggressive and antisocial pathological behaviors (Coccaro, 2012; Coccaro et al., 2007; New et al., 2009). Finally, also the AC is a region recruited during emotion regulation strategies (Grecucci et al., 2013b, c), in particular when considering aggressive impulses control (Coccaro, 2012; Frankle et al., 2005; New et al., 2002; Rosell & Siever, 2015) and socially driven interactions (Etkin et al., 2011; Lavin et al., 2013; Rigoni et al., 2010; Sanfey et al., 2003), such as those involving empathy (Gu et al., 2010; Lamm et al., 2011; Lavin et al., 2013; van Veen & Carter, 2002). Therefore, this region could be involved in the disposition to control the own anger feelings to avoid angry outbursts potentially harmful for others.

In particular, our results show that as anger internalization decreases, the connectivity between these areas increases. This could mean that the inhibitory processes associated with these regions could be characterized by a reciprocal interference that increases as the internalization of anger increases, possibly involving internal conflicts that can be associated with the suppression of anger. For example, the right IFG plays a well-known inhibitory role (Aron et al., 2014); therefore, its weaker inhibition of other right prefrontal brain regions, which is associated with higher anger internalization scores, could explain our results. Instead, the right rostral PFC is particularly involved in switching between stimulus-oriented and stimulus-independent attention (Burgess et al., 2007); therefore, according to our results the right RPFC might be involved in a process of switching attention toward different aspects of a situation eliciting anger, possibly switching between anger externalization and internalization tendencies. Therefore, we can speculate that a reduced connectivity between the right RPFC and other right prefrontal brain regions might be associated with higher anger internalization tendencies and a lower tendency to switches toward externalization motives. However, literature evidence is still lacking on this topic, and further studies are needed to better clarify these points. Indeed, previous methods, such as EEG and stimulatory techniques rely on different brain measures. Nevertheless, our results confirm that Anger-In modulates the strength of right prefrontal brain connectivity.

Last but not least, we did not find an association of rs-FC patterns with BIS/BAS scores. This further analysis was performed to test the hypothesis that externalizing and internalizing anger may be at least partially explained by individual differences in the Behavioral Activation and Inhibition system. According to Gray’s Reinforcement Sensitivity Theory, the behaviors involved in reward sensitivity and in the avoidance of punishments are coordinated by the presence of two self-regulation systems: the Behavioral Activation System (BAS) and the Behavioral Inhibition System (BIS) (Gray, 1990). The BAS is responsible of the appetitive motives’ regulation (Gray, 1990; Leone, et al., 2002). In contrast, the BIS operates in avoiding aversive events (punishments) and in the detection of anxiety-producing stimulus (Gray, 1990; Leone, et al., 2002). Scholars have hypothesized an association of the left lateralized frontal activity with trait BAS (Amodio et al., 2008; Coan & Allen, 2003; Harmon-Jones & Allen, 1997; Sutton & Davidson, 1997; Watson et al., 2016) and of the right lateralized frontal activity with BIS (Sutton & Davidson, 1997; Watson et al., 2016). Additionally, Smits and Kuppens (2005) have demonstrated a correlation between anger externalization and internalization inclinations, with BAS and BIS scores respectively. However, in our study we did not find any significant relation between BIS/BAS and rs-FC prefrontal patterns, even though a correlation between BAS and Anger-Out emerged. This result suggests that the prefrontal asymmetry found in our study is specific for externalizing and internalizing anger, whereas there is no significant association with activation or inhibition behavioral motives. Furthermore, these results are in line with a recent update of the prefrontal asymmetry hypothesis based on the revised Reinforcement Sensitivity Theory (Gray & McNaughton, 2000). Lacey et al. (2020) proposed that the right prefrontal activity is not simply associated with the BIS, but with two systems: on one hand, the Fight Flight Freeze System (FFFS) in response to aversive stimuli and threats, and on the other hand the revised-BIS, that is activated by conflicts of different motivations associated with the BAS and the FFFS. Therefore, the right PFC would not simply be associated with withdrawal motivation (as initially hypothesized) but with a conflict between different motives, associated to what as been called the revised-BIS. In particular, it has been proposed that this revised-BIS is responsible of an effortful control or suppression of motivation, especially when associated with approach behaviors (Lacey et al., 2020). Our results support this hypothesis; on one hand there is anger externalization that is the expression of the approach motivation characterizing this emotion, which is associated with left prefrontal activity or connectivity (and correlates with BAS scores); on the other hand there is anger internalization that is the suppression of the approach motivation characterizing this emotion, which is associated with right prefrontal activity or connectivity. Therefore, the association between these connectivity patterns and more specific affective components, rather than simple approach and withdrawal motives, could explain the reason why we did not find associations between BIS/BAS and prefrontal asymmetry. Following these considerations, the left prefrontal connectivity could be associated with the expression of the natural motive elicited by anger, i.e., aggression and anger externalization; on the other hand, the internalization of this emotion could be an outcome of conflicts between different motives (i.e., externalization vs. suppression of anger) associated with the right prefrontal connectivity.

## Conclusions

This study confirms and extends previous results by showing for the first time two specific lateralized circuits of prefrontal brain regions and their connectivity patterns responsible for individual tendencies in externalizing or internalizing anger. In line with previous studies, we found a left lateralized prefrontal pattern of rs-FC for externalizing anger (Anger-Out), including the left IFG and the left FEF. On the other hand, we found a right lateralized prefrontal pattern for internalizing anger (Anger-In), including the rostral and lateral PFC, the OFC, the FP, the superior, middle and inferior FG and the AC.

Furthermore, the methodological approach used in the present study has some notable remarks. To our knowledge, this is the first attempt to understand the link between frontal asymmetry and anger from an rs-FC point of view. Also, it has been shown that cognitive task performance and task-based MRI activity have poor test-retest reliability (Elliott et al., 2020). Rs activity and questionnaire data may represent more reliable sources of evidence by capturing more stable psychological and neural features (Elliott et al., 2020). Notably, such approach takes into account individual differences across subjects rather than collapsing differences across conditions and levelling out precious information as usually done in task-based studies.

Even if future research in clinical populations is needed, we suggest that our findings could be helpful to set up future treatments for anger dysregulation with noninvasive brain stimulation techniques. TMS and tDCS can be used to decrease the left prefrontal activity (or increase the right prefrontal activity) for individuals with excessive anger externalization and aggression tendencies (such as antisocial personality disorder, narcissistic personality disorder, mania states of bipolar disorder, borderline personality disorder) (Allen et al., 1993; Beeney et al., 2014; Dadomo et al., 2018; De Panfilis et al., 2019; Grecucci et al., 2020; Kano et al., 1992; Novaco, 2010). On the other hand, the usage of these stimulatory techniques to decrease the right prefrontal activity (or increase the left prefrontal activity) can be taken into account for individuals prone to excessively internalize anger (such as anxiety disorders and depression) (Davidson et al., 2000; Telzer et al., 2008).

Despite the merits, our study has some limitations. First, this study used a different methodology compared with previous studies from which the anger asymmetry hypothesis was derived. This reason also led us to limit our interpretations of the specific connectivity patterns that emerged from our results, because more studies are needed to better understand them. Although results seem to converge with previous findings, they may be taken with caution and additional evidence should be provided by the use of magnetic resonance imaging. For example, a task-based fMRI experiment could add additional information on the asymmetry we found at the level of rs-FC. Another limitation regards the use of healthy subjects only. An intriguing hypothesis is that specific classes of patients may display differences in rs-FC when considering their tendencies to abnormally externalize or internalize anger. Future studies may want to address this question in order to improve the treatment of such populations.
